# Safety, Pharmacokinetics, and Drug-Drug Interaction Potential of Intravenous Durlobactam, a β-Lactamase Inhibitor, in Healthy Subjects

**DOI:** 10.1128/AAC.00071-20

**Published:** 2020-06-23

**Authors:** Jason D. Lickliter, Kenneth Lawrence, John O’Donnell, Robin Isaacs

**Affiliations:** a Nucleus Network, Melbourne, Australia; b Tetraphase Pharmaceuticals, Watertown, Massachusetts, USA; c Entasis Therapeutics, Inc., Waltham, Massachusetts, USA

**Keywords:** β-lactamases, drug interactions, pharmacokinetics

## Abstract

Durlobactam (DUR; also known as ETX2514) is a novel β-lactamase inhibitor with broad activity against Ambler class A, C, and D β-lactamases. Addition of DUR to sulbactam (SUL) *in vitro* restores SUL activity against clinical isolates of Acinetobacter baumannii. The safety and pharmacokinetics (PK) of DUR alone and with SUL and/or imipenem-cilastatin (IMI-CIL) were evaluated in healthy subjects. This was a randomized, placebo-controlled study. In part A, subjects, including a cohort of elderly subjects (which received DUR at 1 g), received single ascending doses of DUR ranging from 0.25 to 8 g. In part B, multiple ascending doses of DUR ranging from 0.25 to 2 g were administered every 6 h (q6h) for 29 doses. In parts C and D, the drug-drug interaction (DDI) potential, including the safety, of DUR (1 g) with SUL (1 g) and/or IMI-CIL (0.5/0.5 g) was investigated after single and multiple doses. Plasma and urine concentrations of DUR, SUL, and IMI-CIL were determined. Among 124 subjects, DUR was generally safe and well tolerated when it was administered either alone or in combination with SUL and/or IMI-CIL. After single and multiple doses, DUR demonstrated linear dose-proportional exposure across the studied dose ranges. Renal excretion was a predominant clearance mechanism. No drug-drug interaction potential between DUR and SUL and/or IMI-CIL was identified. SUL-DUR at 1 g (of each component) administered q6h with a 3-h intravenous (i.v.) infusion is under development for the treatment of serious infections due to A. baumannii. (This study has been registered at ClinicalTrials.gov under identifier NCT02971423.)

## Abstract (precis)

Durlobactam (DUR; also known as ETX2514) is a novel β-lactamase inhibitor with broad activity against Ambler class A, C, and D β-lactamases. Addition of DUR to sulbactam (SUL) *in vitro* restores SUL activity against clinical isolates of Acinetobacter baumannii. The safety and pharmacokinetics (PK) of DUR alone and with SUL and/or imipenem-cilastatin (IMI-CIL) were evaluated in healthy subjects. This was a randomized, placebo-controlled study. In part A, subjects, including a cohort of elderly subjects (which received DUR at 1 g), received single ascending doses of DUR ranging from 0.

## TEXT

Acinetobacter baumannii belongs to a cluster of bacterial species referred to as the A. baumannii-A. calcoaceticus complex (ABC), the members of which are associated with serious infections, including hospital-acquired and ventilator-associated bacterial pneumonia and bloodstream, wound, and complicated urinary tract infections (cUTIs) (1, 2). Multidrug-resistant (MDR) isolates account for up to two-thirds of ABC infections (3–5), which are associated with high rates of morbidity (1, 6–9) and mortality rates of 50% or higher (1, 10–12). The rate of multidrug resistance among ABC isolates is increasing, with rates exceeding 50% in many parts of the world (4, 13). As a consequence, an urgent need exists to identify new antimicrobial agents to treat serious ABC infections (14, 15).

Durlobactam (DUR; previously ETX2514) is a novel, diazabicyclooctenone β-lactamase inhibitor (BLI) that potently inhibits class A, C, and D β-lactamases (16–18). DUR demonstrates *in vitro* activity against some *Enterobacteriaceae* but has no *in vitro* activity against ABC. Sulbactam (SUL), a BLI with potent activity against class β-lactamases, exhibits *in vitro* activity against ABC, but its use has been limited by increasing resistance (8). In preclinical studies, the combination of sulbactam-durlobactam (SUL-DUR) exhibited potent *in vitro* and *in vivo* activity against ABC isolates, including carbapenem-resistant ABC and colistin-resistant isolates (17, 19–22).

SUL-DUR is being developed for the treatment of infections caused by ABC isolates, including MDR and carbapenem-resistant isolates. The first-in-human phase 1 study described here was undertaken to evaluate the safety and pharmacokinetics (PK) of DUR after single and multiple ascending doses and the drug-drug interaction (DDI) potential of DUR when administered alone and in combination with SUL and/or imipenem (IMI)-cilastatin (CIL). In addition, the safety and tolerability profiles of DUR coadministered with SUL and IMI-CIL were evaluated after 11 days of dosing. The PK of DUR after single and multiple ascending intravenous (i.v.) doses and of DUL in combination with SUL have also been evaluated in healthy subjects and those with renal impairment as well as subjects undergoing bronchial alveolar lavage (23, 24).

## RESULTS

A total of 124 subjects (94 receiving DUR, 30 receiving placebo) received ≥1 dose of study drug or placebo. In part B, 32 subjects were randomized, but 1 was discontinued because of an infusion site reaction and somnolence. One subject in part D completed the study but was lost to follow-up. All 124 subjects were included in the safety population, and all 94 who received DUR were included in the PK population. Two other subjects completed all study assessments, but the study drug was discontinued for somnolence and nausea (*n* = 1), which occurred on days 1 and 2 and both of which resolved on day 2, and an anaphylactic reaction due to Brazil nut allergy, which occurred after eating a desert containing Brazil nuts on day 6 and which resolved on the same day (*n* = 1). The characteristics of the subjects were generally comparable across the cohorts, except for a higher mean age in the cohort of elderly subjects (Tables 1 and 2).

**TABLE 1:** Baseline characteristics of subjects in the part A single-dose study

Characteristic	Value for subjects receiving^*a*^:								
	Placebo (*n* = 16)	Durlobactam							
		Cohort 1, 0.25 g (*n* = 6)	Cohort 2, 0.5 g (*n* = 6)	Cohort 3, 1.0 g (*n* = 6)	Cohort 4, 1.0 g (*n* = 6)	Cohort 5, 2.0 g (*n* = 6)	Cohort 6, 4.0 g (*n* = 6)	Cohort 7, 8.0 g (*n* = 6)	Cohort 8,^*b*^ 1.0 g (*n* = 6)
Age (yr)									
Mean ± SD	33 ± 16	24 ± 4	31 ± 13	31 ± 13	31 ± 12	31 ± 8	21 ± 2	25 ± 3	70 ± 3
Range	20–74	19–31	20–54	18–54	21–54	23–46	19–24	21–28	66–74
No. (%) of male subjects	11 (70)	3 (50)	3 (50)	3 (50)	5 (83)	3 (50)	5 (83)	2 (33)	5 (83)
No. (%) of Hispanic or Latino subjects	2 (13)	0	2 (33)	0	0	0	0	0	0
No. (%) of subjects by race									
White	15 (94)	4 (67)	3 (50)	5 (83)	6 (100)	4 (67)	2 (33)	5 (83)	5 (83)
Asian	0	1 (17)	1 (17)	1 (17)	0	2 (33)	1 (17)	1 (17)	1 (17)
Black	1 (6)	0	0	0	0	0	2 (33)	0	0
Other	0	1 (17)	2 (33)	0	0	0	1 (16)	0	0
Mean ± SD wt (kg)	77 ± 13	66 ± 14	65 ± 7	67 ± 8	72 ± 12	81 ± 17	74 ± 17	68 ± 16	85 ± 10
Mean ± SD BMI^*c*^ (kg/m^2^)	25 ± 3.4	22 ± 2.7	22 ± 1.7	23 ± 1.6	24 ± 2.7	27 ± 3.1	24 ± 2.9	23 ± 3.5	27 ± 2.4

a Cohort 4 received a 2-h i.v. infusion; all other cohorts received a 3-h i.v. infusion.

b Cohort 8 consisted of elderly subjects >65 years of age.

c BMI, body mass index.

**TABLE 2:** Baseline characteristics of subjects in parts B, C, and D

Characteristic	Value for subjects in^*a*^:										
	Part B					Part C				Part D, cohort 15	
	Placebo (*n* = 8)	Durlobactam				Cohort 13		Cohort 14			
		Cohort 9, 0.25 g (*n* = 6)	Cohort 10, 0.5 g (*n* = 6)	Cohort 11, 1.0 g (*n* = 6)	Cohort 12, 2.0 g (*n* = 6)	DUR at 1 g + SUL (*n* = 6)	Placebo + SUL at 1 g (*n* = 2)	DUR at 1 g + IMI-CIL (*n* = 6)	Placebo + IMI-CIL (*n* = 2)	DUR at 1 g + SUL + IMI-CIL (*n* = 10)	Placebo + SUL + IMI-CIL (*n* = 2)
Age (yr)											
Mean ± SD	30 ± 1	28 ± 5	32 ± 5	25 ± 3	30 ± 6	27 ± 7	29 ± 4	24 ± 3	27 ± 1	28 ± 8	36 ± 6
Range	29–33	19–33	24–38	23–30	24–40	20–40	26–32	21–29	26–27	21–44	32–40
No. (%) of male subjects	6 (75)	3 (50)	3 (50)	4 (67)	5 (83)	4 (67)	1 (50)	3 (50)	1 (50)	7 (70)	2 (100)
No. (%) of Hispanic or Latino subjects	0	0	1 (17)	1 (17)	0	0	0	0	0	0	0
No. (%) of subjects by race											
White	6 (75)	5 (83)	4 (67)	5 (83)	5 (83)	5 (83)	0	5 (83)	2 (100)	9 (90)	1 (50)
Asian	1 (13)	1 (17)	1 (17)	0	0	1 (17)	2 (100)	0	0	1 (10)	1 (50)
Black	0	0	1 (17)	0	0	0	0	0	0	0	0
Other	1 (13)	0	0	1 (17)	1 (17)	0	0	1 (17)	0	0	0
Mean ± SD wt (kg)	80 ± 13	64 ± 9	76 ± 13	73 ± 10	79 ± 17	81 ± 17	74 ± 17	68 ± 16	85 ± 10	77 ± 8	81 ± 9
Mean ± SD BMI^*b*^ (kg/m^2^)	25 ± 3.5	23 ± 2.1	25 ± 3.5	24 ± 3.4	25 ± 4.3	27 ± 3.1	24 ± 2.9	23 ± 3.5	27 ± 2.4	24 ± 2.3	27 ± 1.6

a In parts C and D, the dose of DUR and SUL was 1 g each and the dose of IMI-CIL was 0.5 g.

b BMI, body mass index.

### Part A: single ascending dose.

**FIG 1:**
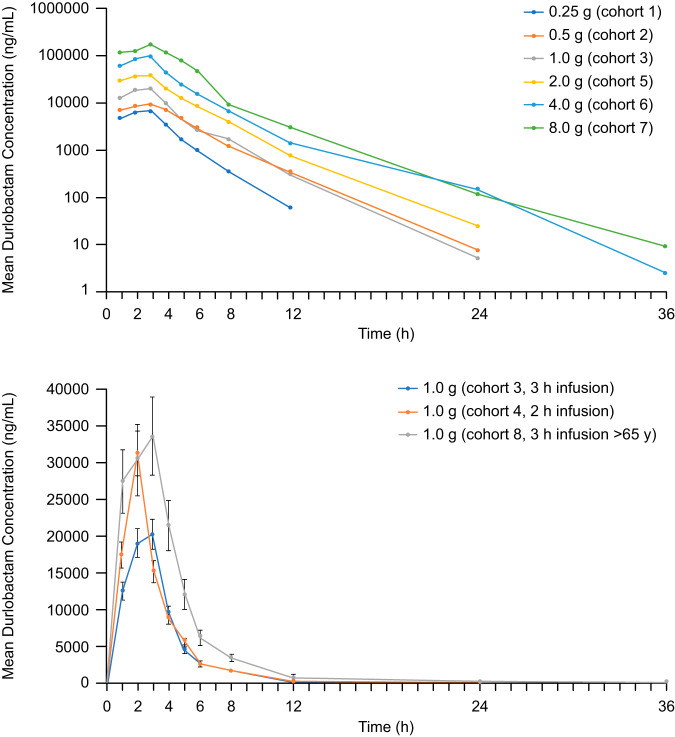
Mean DUR plasma concentrations for cohorts, 1, 2, 3, 5, 6, and 7 (top) and cohorts 3, 4, and 8 (bottom) during the part A single-dose phase.

**FIG 2:**
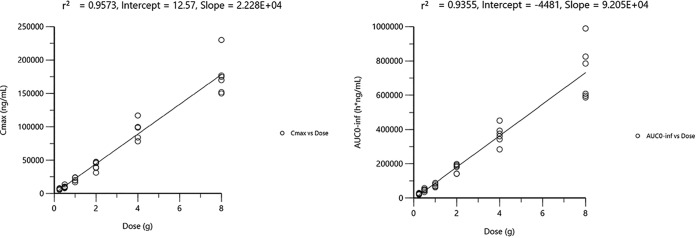
AUC_0–inf_ and *C*_max_ versus dose for the 3-h infusion in cohorts 1, 2, 3, 5, 6, and 7. Rsq, *R* squared.

DUR demonstrated a consistent PK profile and a linear increase in plasma concentrations (Fig. 1) across the range of doses of from 0.25 g to 8 g, with the mean half-life (*t*_1/2_) ranging from 1.5 to 2.8 h (Table 3). A dose-proportional increase in exposure (peak plasma concentration [*C*_max_] and area under the plasma concentration-time curve [AUC]) was observed with increasing doses (Fig. 2). In cohort 4, in which DUR was administered with a 2-h infusion, *C*_max_ was increased approximately 50% compared with that achieved with a 3-h infusion. In cohort 8, consisting of elderly subjects administered DUR at 1 g, *C*_max_ was increased 1.8-fold and AUC was increased 2-fold compared with the values for younger subjects (Fig. 1). The predominant clearance mechanism for DUR was renal excretion (∼50% of intact drug was renally excreted). DUR demonstrated lower total and renal clearance in the cohort of elderly subjects (cohort 8) than in the younger subjects, consistent with renal clearance being the predominant mechanism of elimination (see Table S1 in the supplemental material).

**TABLE 3:** PK parameters for DUR in the part A single-ascending-dose study^*a*^

Cohort	Dose (g)	*t*_1/2_ (h)	*T*_max_ (h)^*b*^	*C*_max_ (μg/ml)	AUC_0–24_ (μg · h/ml)	AUC_0–_*_t_* (μg · h/ml)	AUC_0–inf_ (μg · h/ml)	CL (liters/h)	*V*_ss_ (liters)	*k*_el_ (liter/h)
1	0.25	1.5 ± 0.1	3.0	6.9 ± 1.0	25.5 ± 3.7	25.4 ± 3.7	25.5 ± 3.7	10.0 ± 1.6	16.6 ± 3.6	0.5 ± 0.04
2	0.5	2.0 ± 0.4	3.0	9.9 ± 2.0	47.7 ± 7.5	47.4 ± 7.7	47.8 ± 7.6	10.7 ± 1.9	25.8 ± 2.9	0.4 ± 0.07
3	1.0	2.0 ± 0.3	3.0	20.7 ± 2.8	76.1 ± 10.5	75.6 ± 10.4	76.1 ± 10.5	13.4 ± 1.9	25.7 ± 5.0	0.4 ± 0.04
4^*c*^	1.0	2.2 ± 0.2	2.0	31.3 ± 6.9	100.8 ± 28.9	100.7 ± 29.0	100.9 ± 28.9	10.6 ± 2.7	22.7 ± 6.4	0.3 ± 0.04
5	2.0	2.2 ± 0.2	2.5	41.5 ± 6.4	173.3 ± 25.6	173.3 ± 25.6	173.4 ± 25.6	11.8 ± 1.9	24.3 ± 3.2	0.3 ± 0.03
6	4.0	2.7 ± 0.6	3.0	96.2 ± 13.6	367.3 ± 55.1	367.5 ± 55.0	368.1 ± 55.9	11.1 ± 1.8	22.4 ± 3.6	0.3 ± 0.05
7	8.0	2.8 ± 0.4	3.0	175.7 ± 29.0	730.0 ± 164.4	731.3 ± 163.4	732.1 ± 162.9	11.4 ± 2.3	28.1 ± 9.5	0.3 ± 0.04
8^*d*^	1.0	2.4 ± 0.3	3.0	37.8 ± 2.5	151.2 ± 14.0	151.2 ± 14.0	151.3 ± 14.1	6.7 ± 0.6	14.5 ± 2.0	0.3 ± 0.03

a Values are means ± standard deviations for 6 subjects in each cohort unless indicated otherwise. AUC_0–24_, area under the concentration-time curve from time zero to 24 h; AUC_0–_*_t_*, area under the concentration-time curve from time zero to the last time point evaluated; AUC_0–inf_, area under the concentration-time curve from time zero extrapolated to infinity; CL, clearance; *C*_max_, peak plasma concentration; *k*_el_, elimination rate constant; *t*_1/2_, elimination half-life; *T*_max_, time to *C*_max_; *V*_ss_, volume of distribution at steady state.

b Values are medians.

c Cohort 4 received a 2-h i.v. infusion; all other cohorts received a 3-h i.v. infusion.

d Cohort 8 consisted of elderly subjects >65 years of age.

### Part B: multiple ascending dose.

**FIG 3:**
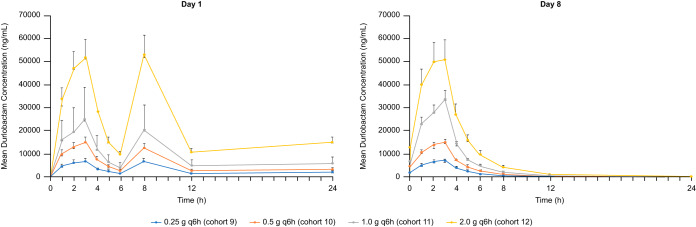
Mean ± standard deviation plasma concentrations for DUR on day 1 and day 8 during the part B multiple-dose phase.

The PK profile of DUR observed after multiple ascending doses of 0.25 g to 2.0 g every 6 h (q6h) for 8 days was generally comparable to that observed after single doses, with minimal accumulation after multiple dosing at day 8 relative to that at day 1 (Table 4). A dose-proportional increase in exposure (*C*_max_ and area under the concentration-time curve from time zero to the end of the dosing period [AUC_0–tau_]) was observed across the dose range (Fig. 3). DUR demonstrated a dose-related increase in fractional excretion on day 1 and day 8 (Table S2).

**TABLE 4:** PK parameters for DUR in the part B multiple-ascending-dose study^*a*^

Cohort	Dose (g)	Day	*t*_1/2_ (h)	*T*_max_ (h)^*b*^	*C*_max_ (μg/ml)	AUC_0–tau_ (μg · h/ml)	CL (liters/h)	*V*_ss_ (liters)	Accum. index
9	0.25	1	ND	3.0	6.9 ± 1.4	23.1 ± 4.9	ND	ND	ND
		8	1.9 ± 0.4	2.5	7.5 ± 1.3	26.2 ± 5.2	9.8 ± 1.7	18.5 ± 2.9	1.1 ± 0.07
10^*c*^	0.5	1	ND	3.0	14.9 ± 2.2	50.3 ± 9.0	ND	ND	ND
		8	2.6 ± 0.1	3.0	14.8 ± 1.1	53.4 ± 4.8	9.4 ± 0.9	18.1 ± 1.8	1.3 ± 0.01
11^*d*^	1.0	1	ND	2.5	26.9 ± 13.1	79.8 ± 35.9	ND	ND	ND
		8	3.5 ± 0.9	3.0	33.4 ± 6.0	108.7 ± 13.9	9.3 ± 1.1	17.2 ± 2.0	1.5 ± 0.18
12	2.0	1	ND	3.0	51.9 ± 8.0	179.1 ± 29.9	ND	ND	ND
		8	10.1 ± 2.9	3.0	53.3 ± 7.8	192.6 ± 31.2	10.6 ± 1.5	21.8 ± 2.0	3.0 ± 0.69

a Values are means ± standard deviations unless indicated otherwise. Doses were administered q6h via a 3-h i.v. infusion. Accum. index, accumulation index (which is equal to $1/1-e^{-k_{\text{el}}}{{}^{\cdot }}^{\text{tau}}$); AUC_0–tau_, area under the concentration-time curve from time zero to the end of the dosing period; CL, clearance; *C*_max_, peak plasma concentration; ND, not determined; *t*_1/2_, elimination half-life; *T*_max_, time to *C*_max_; *V*_ss_, volume of distribution at steady state.

b Values are medians.

c Data are for 4 subjects.

d Data are for 5 subjects.

### Part C: DUR-SUL drug-drug interaction.

The coadministration of a single dose of DUR and sulbactam (cohort 13) did not alter the PK profile of DUR or SUL (Table 5). Further, coadministration had no effect on the urinary excretion of either DUR or SUL (Table S3). The coadministration of a single dose of DUR with IMI-CIL and/or SUL (cohort 14) had no effect on the PK profile of DUR or IMI-CIL (Table 5). The coadministration of DUR, SUL, and/or IMI-CIL also had no effect on the urinary excretion of DUR, and no changes in urinary parameters were observed for SUL, IMI, or CIL (Table S3).

**TABLE 5:** PK parameters for DUR, SUL, and IMI-CIL alone and after coadministration in part C^*a*^

Drug regimen	*t*_1/2_ (h)	*C*_max_ (μg/ml)	AUC_0–24_ (μg · h/ml)	CL (liters/h)	*V*_ss_ (liters)
Durlobactam PK cohort 13					
DUR, day 1	2.0 ± 0.4	26.9 ± 3.6	104.4 ± 6.6	9.6 ± 0.6	17.6 ± 2.0
DUR + SUL, day 5	2.0 ± 0.4	28.1 ± 2.5	105.4 ± 6.4	9.5 ± 0.6	17.4 ± 1.7
Sulbactam PK cohort 13					
SUL, day 1	1.3 ± 0.1	20.7 ± 0.7	68.5 ± 4.7	14.6 ± 1.0	18.0 ± 1.6
SUL + DUR, day 5	1.3 ± 0.1	22.0 ± 2.7	73.0 ± 6.2	13.8 ± 1.2	18.1 ± 1.7
Durlobactam PK cohort 14					
DUR, day 1	1.8 ± 0.4	30.0 ± 9.2	106.2 ± 31.5	9.9 ± 2.1	15.9 ± 2.6
DUR + IMI-CIL, day 5	1.7 ± 0.3	26.6 ± 5.1	103.2 ± 22.6	9.9 ± 1.8	15.4 ± 1.9
DUR + IMI-CIL + SUL, day 8	1.9 ± 0.4	30.4 ± 7.8	115.4 ± 28.5	9.0 ± 1.8	14.9 ± 2.3
Sulbactam PK cohort 14					
DUR + SUL+ IMI-CIL, day 8	1.3 ± 0.1	23.7 ± 6,1	80.6 ± 21.2	13.0 ± 2.6	15.2 ± 2.7
SUL+ IMI-CIL, day 8	1.3 ± 0.1	24.0 ± 2.0	81.7 ± 5.2	12.3 ± 0.8	15.4 ± 1.5
Imipenem PK cohort 14					
IMI-CIL, day 3	1.2 ± 0.1	34.4 ± 5.3	43.7 ± 9.2	11.8 ± 2.1	14.3 ± 2.2
IMI-CIL + DUR, day 5	1.2 ± 0.2	31.3 ± 2.3	42.2 ± 5.6	12.0 ± 1.4	15.2 ± 1.2
IMI-CIL + SUL, day 8	1.2 ± 0.1	32.7 ± 4.0	45.7 ± 5.1	11.0 ± 1.2	13.9 ± 1.0
Cilastatin PK cohort 14					
IMI-CIL, day 3	1.2 ± 0.2	46.0 ± 8.3	49.6 ± 9.8	10.4 ± 1.8	9.6 ± 1.4
IMI-CIL + DUR, day 5	1.2 ± 0.2	44.0 ± 6.1	47.1 ± 7.2	10.8 ± 1.8	9.9 ± 1.2
IMI-CIL + DUR + SUL, day 8	1.2 ± 0.2	40.7 ± 4.9	46.9 ± 6.6	10.9 ± 1.6	10.4 ± 0.9

a Values are means ± standard deviations. The dose of DUR and SUL was 1 g each; the dose of IMI-CIL was 0.5 g. AUC_0–24_, area under the concentration-time curve from time zero to 24 h; CL, clearance; *C*_max_, peak plasma concentration; *t*_1/2_, elimination half-life; *V*_ss_, volume of distribution at steady state.

### Part D: multiple-dose administration of DUR, SUL, and IMI-CIL.

After administration of DUR, *C*_max_, the time to *C*_max_ (*T*_max_), and AUC_0–tau_ were unchanged between days 1 and 11 (Table 6), indicating no apparent accumulation. In addition, the DUR *C*_max_ and *T*_max_ obtained following multiple doses in combination with SUL and/or IMI-CIL were comparable to those obtained following single doses of DUR. The PK profiles of SUL, IMI, and CIL were unchanged between days 1 and 11, consistent with no accumulation; the accumulation index was approximately 1. No apparent change in urinary parameters was observed for any study drug with coadministration (Table S4).

**TABLE 6:** PK parameters for DUR, SUL, IMI, and CIL on day 1 and day 11 after coadministration in part D^*a*^

Cohort	day	*t*_1/2_ (h)	*T*_max_^*b*^ (h)	*C*_max_ (μg/ml)	AUC_0–tau_ (μg · h/ml)	CL (liters/h)	*V*_ss_ (liters)	Accum. index
DUR	1	ND	2.8	27.1 ± 1.3	91.8 ± 5.9	ND	ND	ND
	11	4.3 ± 3.0	2.4	28.1 ± 8.6	96.3 ± 11.6	10.5 ± 1.2	21.4 ± 4.9	1.6 ± 0.7
SUL	1	ND	2.7	23.9 ± 1.3	76.7 ± 5.8	ND	ND	ND
	11	2.0 ± 1.0	2.6	22.4 ± 6.1	67.9 ± 7.6	14.5 ± 1.5	20.3 ± 5.8	1.2 ± 0.21
IMI	1	ND	0.5	24.7 ± 5.2	35.7 ± 4.4	ND	ND	ND
	11	1.5 ± 0.3	0.5	24.1 ± 5.3	33.1 ± 5.4	15.5 ± 2.2	21.7 ± 3.8	1.1 ± 0.04
CIL	1	ND	0.5	38.6 ± 6.0	45.6 ± 7.0	ND	ND	ND
	11	1.7 ± 0.3	0.5	38.0 ± 5.6	39.4 ± 7.2	13.1 ± 2.4	13.9 ± 2.1	1.1 ± 0.04

a The values are means ± standard deviations for 10 subjects. Doses were administered q6h via a 3-h i.v. infusion. The dose of DUR and SUL was 1 g each; the dose of IMI-CIL was 0.5 g. Accum. index, accumulation index; AUC_0–tau_, area under the concentration-time curve from time zero to the end of the dosing period; CL, clearance; *C*_max_, peak plasma concentration; ND, not determined; *t*_1/2_, elimination half-life; *T*_max_, time to *C*_max_; *V*_ss_, volume of distribution at steady state.

b Values are medians.

### Safety and tolerability.

DUR was generally safe and well tolerated. No dose-related trends were observed for any of the treatment-emergent adverse events (AEs), with the possible exception of phlebitis and catheter site phlebitis in subjects receiving multiple doses. Following a detailed review, there was no consistent pattern of local infusion site reactions to suggest a specific concern. When DUR was coadministered with SUL and IMI-CIL for 11 days (part D), the tolerability profile of DUR was not substantially different from that achieved with multiple dosing of DUR alone (part B). The most common treatment-emergent AEs (found in ≥5% of subjects) were headache (DUR group, 13.8%; placebo group, 13.3%) and catheter site phlebitis (DUR group, 8.5%; placebo group, 3.3%). The most common drug-related AEs (found in ≥3% of subjects) were headache (DUR group, 10.6%; placebo group, 10.0%) and catheter site phlebitis (DUR group, 5.3%; placebo group, 0%) (Table 7).

**TABLE 7:** Incidence of drug-related AEs occurring in >1% of subjects

Adverse event	No. (%) of subjects			
	All subjects receiving DUR (*n* = 94)	All subjects receiving placebo (*n* = 30)	All subjects receiving DUR + SUL and IMI-CIL (*n* = 10)	All subjects receiving placebo + SUL and IMI-CIL (*n* = 2)
Abdominal pain	0	0	0	1 (50.0)
Catheter site phlebitis	5 (5.3)	0	0	0
Dizziness	4 (4.3)	1 (3.3)	0	0
Dysgeusia	2 (2.1)	0	2 (20.0)	0
Headache	10 (10.6)	3 (10.0)	2 (20.0)	0
Musculoskeletal stiffness			1 (10.0)	0
Nasal congestion	2 (2.1)	0	0	0
Nausea	2 (2.1)	1 (3.3)	0	0
Pain in extremity	1 (1.1)	1 (3.3)	0	0
Phlebitis	2 (2.1)	0	0	0
Polydipsia	0	0	1 (10.0)	0
Pruritus	2 (2.1)	1 (3.3)	0	0
Somnolence	1 (1.1)	1 (3.3)	0	0
Upper respiratory infection	1 (1.1)	1 (3.3)	0	0
Vulvovaginal candidiasis	2 (2.1)	0	1 (10.0)	0

In part B, one subject in the cohort receiving DUR at 0.5 g q6h discontinued therapy for drug-related mild-moderate somnolence (mild) and nausea (moderate). The onset was on days 1 and 2, but both events resolved by day 2. Both events were considered possibly or probably related to therapy. In the same cohort receiving DUR at 0.5 g q6h, one subject with a known nut allergy experienced a serious AE, an anaphylactic reaction to Brazil nuts, which was considered unrelated to therapy. One subject in the cohort receiving DUR at 1 g q6h discontinued for an infusion reaction (moderate, considered unrelated to therapy) and somnolence (mild, unrelated). Both events occurred on day 1 and resolved by day 1. The infusion site reaction was a systemic response rather than catheter related.

No clinically significant changes in clinical laboratory values, vital signs, or electrocardiogram (ECG) findings were observed in any subject. Decreases in mean neutrophil counts were observed in subjects receiving DUR in part B, but mean neutrophil count values remained within the reference range at all time points, and these changes were not considered clinically significant by the study investigator.

## DISCUSSION

In the single-dose phase, single doses of DUR administered across the dose range of from 0.25 g to 8 g via a 3-h i.v. infusion demonstrated generally linear, dose-proportional exposure. In the single cohort that received DUR at 1 g with a 2-h infusion, *C*_max_ increased by approximately 50%. In the cohort of elderly subjects, exposure was increased by 100%, the half-life was prolonged, and renal clearance and the volume of distribution (*V*) were reduced by approximately 50%; these observations are consistent with renal clearance as a predominant mechanism of elimination. In the multiple-dose phase, DUR demonstrated a linear dose-proportional exposure across the dose range of from 0.25 g to 2 g infused over 3 h q6h. Minimal accumulation of DUR was observed up to day 8, which is consistent with a short half-life. No significant effects on the PK profile of any study drug were observed with the coadministration of DUR and SUL or of DUR and IMI-CIL. DUR was safe and well tolerated at single doses up to 8 g and at multiple doses up to 2 g q6h for up to 8 days. The safety profile of DUR was unchanged when it was coadministered as a single dose with SUL, IMI-CIL, and SUL plus IMI-CIL. DUR was associated with a low rate of discontinuation because of AEs, and there were no serious AEs.

The PK results from this study are consistent with those from other studies in healthy subjects in which DUR administered alone and in combination with SUL and/or IMI-CIL was well tolerated, including in subjects with various degrees of renal impairment (23, 24). In these studies, the DUR half-life ranged from 1.4 to 2.3 h, *C*_max_ ranged from 27.0 to 33.4 μg/ml, and AUC ranged from 102 to 110 μg · h/ml (23, 24). The PK results are also consistent with those in patients with cUTIs, where no accumulation of DUR was observed following a 7-day dosing regimen (25), which is consistent with the results of the multiple-ascending-dose portion of this study in healthy subjects. In the renal impairment study (23), no AEs were reported in healthy subjects, and only single AEs were reported in subjects undergoing assessment of DUR pulmonary concentrations (24). In a phase 2 study of patients with cUTI, only 2 patients discontinued because of AEs, no serious AEs were reported, and the most common AEs were headache, nausea, diarrhea, and vascular pain, occurring in 3.8% to 5.7% of patients (25). While renal excretion predominates as a major clearance mechanism for DUR, non-cytochrome P450-mediated hydrolytic cleavage of the diazabicyclooctenone core and bioconjugated metabolites account for the remaining clearance of DUR. Accounting for protein in human plasma (protein binding = 0.9) and a glomerular filtration rate (GFR) in healthy subjects of ∼90 ml/min, the renal clearance of DUR exceeds what would be expected via filtration and suggests an active (secretory) component. An *in vitro* assessment of transporter interactions with DUR has confirmed an affinity of the substrate for the renal transporter OAT1 (data not published), confirming a role for active transport in the renal excretion of DUR.

SUL-DUR is undergoing clinical development for the treatment of serious infections due to ABC pathogens, including the treatment of hospitalized patients with pneumonia or bacteremia. Administration of SUL-DUR in an ongoing phase 3 trial (ClinicalTrials.gov identifier NCT03894046) is utilizing IMI-CIL as the background carbapenem therapy, as these serious Gram-negative bacterial infections are often polymicrobial in nature. Confirming the lack of a potential for a DDI of SUL-DUR with IMI-CIL was an important component of the present study. Infections caused by ABC are associated with high rates of multidrug resistance, increased rates of morbidity, and extended hospitalization relative to infections caused by other pathogens (1, 3, 26). Currently, colistin is the only antibiotic that demonstrates consistent antimicrobial activity against ABC pathogens (27). Nevertheless, mortality rates are approximately 40% among patients with hospital-acquired or ventilator-associated pneumonia who are treated with colistin-based antibiotic regimens (27). Treatment with colistin is further complicated by dose-related toxicity, especially nephrotoxicity, which occurs in 40% or more of patients (28, 29). Thus, a critical unmet need remains for novel and safer treatment approaches for treating serious infections due to ABC pathogens.

Based on the PK modeling conducted with this study and the renal impairment study (23), the dosage regimen of SUL-DUR for optimal target attainment against ABC is 1 g (of each component) administered q6h with a 3-h i.v. infusion (30). A global phase 3 study is evaluating the efficacy and safety of SUL-DUR for treating serious infections due to ABC in hospitalized patients.

## MATERIALS AND METHODS

The study enrolled subjects at a single clinical site in Australia between September 2016 and August 2017. The study was conducted in accordance with the Declaration of Helsinki and good clinical practices. The study protocol was approved by an institutional review board (Nucleus Network, Alfred Hospital Ethics Committee, Melbourne, Australia, John J. McNeil, Chair). All subjects provided written informed consent prior to any study procedure. This study was registered at ClinicalTrials.gov under identifier NCT02971423.

### Study design.

This was a 4-part, double-blind, placebo-controlled study (15 cohorts) of DUR administered as a 3-h i.v. infusion (except for cohort 4, in which a 2-h i.v. infusion was evaluated) (see Table S5 in the supplemental material). Part A was a single-ascending-dose escalation phase that included eight cohorts (each with 6 subjects receiving active drug and 2 subjects receiving placebo), including one cohort (cohort 4) in which DUR was administered with a 2-h i.v. infusion and one cohort (cohort 8) of elderly subjects aged ≥65 years who received DUR at 1 g via a 3-h i.v. infusion. Part B was a multiple-ascending-dose escalation phase consisting of four cohorts (each with 6 subjects receiving active drug and 2 subjects receiving placebo), in which the subjects received DUR at 0.25, 0.5, 1, or 2 g infused i.v. over 3 h q6h or placebo for 29 doses.

Part C was a crossover drug-drug interaction phase consisting of two cohorts (each with 6 subjects receiving active drug and 2 subjects receiving placebo). Cohort 13 was used for a 2-way single-dose comparison between DUR at 1 g and sulbactam at 1 g. Cohort 14 was used for a 2-way single-dose comparison between DUR at 1 g and IMI-CIL at 0.5/0.5 g. Part D was a repeat-dosing phase of SUL-DUR plus IMI-CIL (10 subjects receiving active drug and 2 subjects receiving placebo). In cohort 15, subjects received 1 g DUR i.v. or placebo and 1 g SUL i.v., which were infused over 3 h, and 1 g DUR i.v. or placebo and 0.5 g IMI-CIL i.v., which were infused over 30 min q6h, for 41 doses.

### Subject selection.

Healthy adult male and female subjects (age range, 18 to 55 years) and a single cohort of elderly subjects (age, ≥65 years) were eligible if they were in good general health, had a body mass index of 18 to 32 kg/m^2^ inclusive, and had no clinically significant medical history. Subjects were required to have clinical laboratory values within normal limits and a negative screen for drugs of abuse, alcohol, hepatitis B surface antigen (HBS Ag), hepatitis C virus antibody (HCV Ab), and human immunodeficiency virus (HIV) at screening. Female subjects were of nonchildbearing potential or used a medically acceptable contraceptive regimen and had a negative serum pregnancy test at screening and a negative urine pregnancy test prior to study drug dosing. Male subjects were surgically sterile or used a medically acceptable contraceptive regimen.

Subjects were excluded because of hypersensitivity or an allergic reaction to any beta-lactam antibiotic, the use of prescription or over-the-counter medications within 7 days of study drug administration, or participation in an investigational drug study within 30 days; if they were current smokers; if they had a history of major organ dysfunction; if they had an infection or underlying medical condition that would interfere with taking the study drug; if they had a history of excessive alcohol intake; or if they had a concomitant disease or condition that could interfere with the conduct of the study.

### Study assessments.

A routine physical examination, determination of vital signs (supine blood pressure, heart rate, respiratory rate, temperature), a 12-lead electrocardiogram (ECG), and clinical laboratory testing (serum chemistry, hematology, urinalysis) were performed at screening, at intervals during the study, and at the 14-day follow-up.

For part A, plasma samples for PK analysis were collected at 30 min prior to the dose and 1, 2, 3, 3.5, 4, 5, 6, 8, 12, 24, 36, and 48 h after the dose, except for cohort 4 (which received the study drug as a 2-h infusion), for which collection occurred at 30 min prior to the dose and 1, 2, 2.5, 3, 4, 5, 6, 8, 12, 24, 36, and 48 h after the dose. For part B, plasma samples were collected on day 1 at 30 min prior to the dose and 1, 2, 3, 3.5, 4, 5, 6 (prior to the next dose), 8, and 12 (prior to the next dose) h; on day 2 and day 4 at 30 min prior to the second dose; and on day 8 at 30 min prior to the final dose and 1, 2, 3, 3.5, 4, 5, 6, 8, 12, 24, 36, and 48 h after the dose.

For part C, for cohort 13, plasma samples were collected 30 min prior to the dose and at 1, 2, 3, 3.5, 4, 5, 6, 8, 12, 24, 36, and 48 (immediately prior to SUL administration) h; then at 1, 2, 3, 3.5, 4, 5, 6, 8, 12, 24, 36, and 48 h (immediately prior to SUL-DUR dose administration); and then at 1, 2, 3, 3.5, 4, 5, 6, 8, 12, 24, 36, and 48 h. For cohort 14, plasma samples were collected 30 min prior to DUR administration and at 1, 2, 3, 3.5, 4, 5, 6, 8, 12, 24, 36, and 48 h (immediately prior to IMI-CIL administration); then at 1, 2, 3, 3.5, 4, 5, 6, 8, 12, 24, 36, and 48 h (immediately prior to SUL and IMI-CIL administration); and then at 1, 2, 3, 3.5, 4, 5, 6, 8, 12, 24, 36, and 48 h. On day 8, samples were collected 30 min prior to DUR, SUL, and IMI-CIL administration and at 1, 2, 3, 3.5, 4, 5, 6, 8, 12, 24, 36, and 48 h after the dose.

For part D, plasma samples were collected on day 1 30 min prior to the dose and at 1, 2, 3, 3.5, 4, 5, 6 (prior to the next dose), 8, and 12 h (prior to the next dose); 30 min prior to the second dose on days 2 and 4; and on day 11 at 30 min prior to the final dose and at 1, 2, 3, 3.5, 4, 5, 6, 8, 12, 24, 36, and 48 h.

For all cohorts, urine was collected from −12 to 0 h prior to the start of infusion and at 0 to 6, 6 to 12, 12 to 24, and 24 to 48 h after start of infusion for determination of the clearance of DUR. In addition, urine was collected at 0 to 6, 6 to 12, 12 to 24, and 24 to 48 h postinfusion on days 8 to 10 for part B; on days 3 to 5 and 5 to 7 for cohort 13 for part C; on days 3 to 5, 5 to 7, and 8 to 10 for cohort 14 for part C; and on days 11 to 13 for part D.

### Determination of DUR, SUL, CIL, and IMI plasma concentrations.

The concentrations of DUR, SUL, CIL, and IMI in plasma were determined by a validated liquid chromatography with tandem mass spectrometry (LC-MS/MS) assay operated in the negative ion mode (Covance method numbers M8351072B, M8356245, and M8356242), performed at Covance (Salt Lake City, UT). A total of 2,360 plasma samples were assayed between October 2016 and January 2018 (Covance bioanalytical report 8355766). A subset of samples was retained for incurred sample assay reproducibility (ISR) testing, and the calculated assay variability values met the acceptance criteria, with at least two-thirds of the repeat results and original results falling within 20% of the mean of the two values. For all analytical runs, fortified calibration standards and quality controls (QCs) met the acceptance criteria of concentrations within ±15% of the nominal concentration (±20% at the lower limit of quantification [LLOQ]).

Clinical samples were processed by protein precipitation with acetonitrile to isolate DUR, SUL, CIL, and IMI. Stable labeled internal standards (ETX2514-13C2-15N2 [AstraZeneca lot no. AZ13572514-015], sulbactam sodium-d2 [Entasis Therapeutics lot no. 1S601], imipenem-d4 [Entasis Therapeutics lot no. 57513-190D1], and cilastatin-13C-15N [Entasis Therapeutics lot no. N70-63]) were utilized to establish sample and calibration analyte/internal standard peak area ratios. The blank control matrix was made up of human plasma (BioreclamationIVT) diluted 1:1 with SigmaFast (reconstituted protease inhibitor cocktail tablet [product S8820; Sigma-Aldrich, St. Louis, MO] in 10 ml of water) for preparation of calibration standards and QC samples. Sample extraction of 50-μl aliquots of clinical samples, fortified standards, and QC samples was completed following addition of 50 μl of the internal standard (250 ng/ml in 50:50 water-acetonitrile). Acetonitrile (400 μl) was added, and the mixture was vortexed for 5 min prior to centrifugation at 3,500 × g for 10 min to separate the precipitated protein from the supernatant. A 100-μl aliquot of the supernatant was transferred to 96-well plates. Samples were diluted further with 400 μl of water, and 5 μl of sample was injected into the LC-MS/MS system for analysis. The standard curves were linear for DUR (mean *r*^2^ ≥ 0.996) and CIL (mean *r*^2^ ≥ 0.997) over a concentration range of 5.0 to 5,000 ng/ml. The standard curves for SUL and IMI fit a quadratic regression (mean *r*^2^, ≥0.997 and ≥0.997, respectively) over a concentration range of 5.0 to 5,000 ng/ml. The respective precision and accuracy for DUR QC samples were 7.9% and −0.7% at 15 ng/ml, 6.9% and −3.0% at 175 ng/ml, 4.2% and 0.5% at 2,000 ng/ml, and 11.4% and −2.8% at 4,000 ng/ml. The respective precision and accuracy for SUL QC samples were 7.9% and −1.3% at 15 ng/ml, 4.7% and −5.1% at 175 ng/ml, 3.5% and −2.0% at 2,000 ng/ml, and 4.6% and −2.8% at 4,000 ng/ml. The respective precision and accuracy for CIL QC samples were 7.9% and 0.0% at 15 ng/ml, 8.0% and 6.3% at 175 ng/ml, 5.0% and 1.5% at 2,000 ng/ml, and 5.6% and −0.3% at 4,000 ng/ml. The respective precision and accuracy for IMI QC samples were 23.2% and −11.3% at 15 ng/ml, 4.9% and −4.6% at 175 ng/ml, 6.4% and −4.5% at 2,000 ng/ml, and 4.1% and −3.5% at 4,000 ng/ml. The LLOQ for all analytes was 5 ng/ml. Reported concentrations were multiplied by a factor of 2 to account for the 1:1 dilution of plasma samples with the SigmaFast protease cocktail solution.

### Study analysis.

The PK parameters for the single-dose arm (part A) were the peak plasma concentration (*C*_max_), the plasma concentration at time *t* (*C_t_*), the time to the peak plasma concentration (*T*_max_), the area under the concentration-time curve from time zero to 24 h (AUC_0–24_), the AUC from time zero to the last time point evaluated (AUC_0–_*_t_*), the AUC from time zero extrapolated to infinity (AUC_0–inf_), elimination rate constant (*k*_el_), elimination half-life (*t*_1/2_), clearance (CL), volume of distribution (*V*), cumulative excretion of unchanged drug in urine (*A_e_*), urinary (renal) clearance (CL_R_), fraction excreted unchanged in urine (*f_e_*), and assessment of dose proportionality. For the multiple-dose arms (parts B and D), additional parameters were the AUC from time zero to the end of the dosing period (AUC_0–tau_) and the accumulation ratio (*R*_0_). AUC was calculated using the log-linear trapezoidal rule. The half-life (*t*_1/2_) was estimated as ln_2_/*k*_el_. Urine PK parameters were most accurately determined from cohorts 6, 7, and 8, for whom data were available to 48 h postdose.

Pharmacokinetic parameters were summarized at each assessment time point using the mean ± standard deviation (SD), the percent coefficient of variation (CV), median, minimum, and maximum. Single-ascending-dose study data were assessed for dose proportionality using *C*_max_ and AUC, and accumulation ratios was determined using repeat-dose cohorts. All plasma concentrations below the limit of quantitation preceding *C*_max_ were set to 0, and all plasma concentrations below the limit of quantitation following *C*_max_ were set to missing. Noncompartmental analysis of PK parameters was performed using Phoenix WinNonlin (v6.4) software to determine PK parameters.

The intent-to-treat (ITT) population included all randomized subjects. The safety population was all randomized subjects who received any study drug. The PK population was all randomized subjects who received any study drug and who had at least one quantifiable plasma concentration for the treatment received.

## Supplementary Material

Supplemental file 1
