# Supplementary material for: Safety, Pharmacokinetics, and Drug-Drug Interaction Potential of Intravenous Durlobactam, a β-Lactamase Inhibitor, in Healthy Subjects
Source: Antimicrob Agents Chemother. 2020 Jun 23;64(7):e00071-20. doi: 10.1128/AAC.00071-20 (PMC7318034; doi:10.1128/AAC.00071-20)
Supplement: Supplemental file 1 [file AAC.00071-20-s0001.pdf]

Supplemental Data

Table S1. Summary of Urine PK Parameters for Durlobactam for Cohorts 6, 7, and 8 (PK Parameters Population)

Cohort	Dose (g)	Statistics	CL_r (mL/hour)	F_e (%)	A_e (mg)
6	4	n	4	4	4
		Mean	7947.08	76.4345	3057.384
		SD	1222.484	11.12174	444.862
		Min	6419.0	63.095	2523.81
		Median	8018.75	77.8635	3114.55
		Max	9331.8	86.916	3476.63
7	8	n	5	5	5
		Mean	6524.34	55.0196	4401.576
		SD	3294.011	25.48131	2038.512
		Min	1173.8	11.525	921.99
		Median	7778.70	58.8180	4705.45
		Max	9848.9	73.565	5885.20
8	1	n	5	5	5
		Mean	3323.60	58.2604	582.604
		SD	1332.995	3.36137	33.613
		Min	1003.2	55.550	555.50
		Median	3962.10	57.3870	573.87
		Max	4170.0	64.009	640.09

A_e: cumulative excretion of unchanged drug in urine; CL_r: urinary clearance; F_e: fraction excreted unchanged in urine; Max: maximum; Min: minimum; n: number of subject in the category; SD: standard deviation.

Table S2. The First 48 Hours: Summary of Urine PK Parameters for Durlobactam (PK Parameters Population)

Cohort	Dose[a] (g)	Statistics	Urine Volume (mL)	F_e (%)	A_e (mg)	CL_r[b] (mL/hour)
9	2.00	n	6	6	6	5
		Mean	4849.3	47.662	953.242	14286.10
		SD	1812.93	12.528	250.571	3464.310
		Median	5408.5	51.57	1031.41	13208.70
10	4.00	n	6	6	6	6
		Mean	5258.8	53.939	2157.551	16338.13
		SD	2586.91	13.869	554.773	4346.491
		Median	5065.5	57.21	2288.24	17459.05
11	8.00	n	6	6	6	5
		Mean	3527.8	56.699	4535.949	21837.96
		SD	1103.93	27.878	2230.256	4233.911
		Median	3498.5	65.06	5204.73	23503.80
12	16.00	n	6	6	6	4
		Mean	5334.7	55.354	8856.594	18449.33
		SD	2248.02	13.973	2235.642	6556.986
		Median	4759.5	49.25	7879.32	18404.50

A_e: cumulative excretion of unchanged drug in urine; CL_r: urinary clearance; F_e: fraction excreted unchanged in urine; n: number of subject in the category; PK: pharmacokinetic; SD: standard deviation.

a. Dose calculated over 48 hours (ie 8 individual doses).

b. In subjects where a complete 48-hour collection interval was not available, CL_r was not calculated.

Table S2. Following the Final Dose: Summary of Urine PK Parameters for Durlobactam (PK Parameters Population)

Cohort	Dose[a] (g)	Statistics	Urine Volume (mL)	F_e (%)	A_e (mg)	CL_r (mL/hour)
9		n	6	6	6	5
	0.250	Mean	1703.500	56.473	141.184	5046.44
	0.000	SD	495.207	25.196	62.990	2916.824
	0.25	Median	1857.50	60.48	151.20	4407.40
10		n	4	4	4	4
	0.500	Mean	1771.750	67.523	337.614	5736.98
	0.000	SD	933.486	13.157	65.784	1271.318
	0.50	Median	1802.00	65.88	329.39	5773.85
11		n	5	5	5	5
	1.000	Mean	1350.400	65.711	657.112	5616.22
	0.000	SD	386.324	11.922	119.221	1483.653
	1.00	Median	1306.00	67.09	670.87	5878.20
12		n	6	6	6	5
	2.000	Mean	1638.333	69.336	1386.730	6460.44
	0.000	SD	606.565	14.686	293.717	1043.593
	2.00	Median	1646.50	63.93	1278.52	6557.80

A_e: cumulative excretion of unchanged drug in urine; CL_r: urinary clearance; F_e: fraction excreted unchanged in urine; n: number of subject in the category; PK: pharmacokinetic; SD: standard deviation.

a. Based on the final dose on Day 8.

Table S3. Cohort 13: Selected Durlobactam and Sulbactam Urine PK Parameters (PK Parameters Population)

	Durlobactam		Sulbactam	
**Parameter**	**Day 1**<br>n = 6	**Day 5**<br>n = 6	**Day 3**<br>n = 5	**Day 5**<br>n = 5
n (%)				
F_e (%)	40.964 (6.869)	49.963 (9.530)	69.189 (5.544)	66.417 (9.630)
A_e (mg)	409.644 (68.690)	499.627 (95.305)	691.885 (55.442)	664.171 (96.295)
CL_r (mL/hour)	NC	4857.93 (1112.552)	10142.88 (821.545)	9169.60 (1610.915)

A_e: cumulative excretion of unchanged drug in urine; CL_r: urinary clearance; F_e: fraction excreted unchanged in urine; n: number of subject in the category; NC: not countable; PK: pharmacokinetic.

Table S3. Cohort 14: Mean (SD) Urine PK Parameters (PK Parameters Population)

	Durlobactam			Sulbactam		Imipenem		
**Parameter**<br>Mean (SD)	**Day 1**<br>n = 6	**Day 5**<br>n = 6	**Day 8**<br>n = 6	**Day 8**<br>n = 5	**Day 3**<br>n = 6	**Day 5**<br>n = 6	**Day 8**<br>n = 6	**Day 3**<br>n = 6
**F_e (%)**	42.664 (10.805)	31.605 (9.774)	19.329 (8.5909)	47.935 (26.668)	50.418 (8.863)	50.711 (8.889)	38.063 (18.808)	84.282 (6.308)
**A_e (mg)**	426.643 (108.055)	316.048 (97.736)	193.288 (85.909)	479.348 (266.683)	252.090 (44.316)	253.557 (44.445)	190.318 (90.042)	421.411 (31.541)
**CL_r (mL/hour)**	NC	3228.15 (1289.87)	2127.60 (283.83)[a]	NC	6100.50 (1843.92)	6140.70 (1429.02)	4192.140 (2212.249)	7964.278 (1140.792)

A_e: cumulative excretion of unchanged drug in urine; CL_r: urinary clearance; F_e: fraction excreted unchanged in urine; n: number of subject in the category; NC: not countable; PK: pharmacokinetic; SD: standard deviation.

a. n = 2.

Table S3. Cohort 14: Day 8 Mean (SD) Imipenem and Cilastatin Urine PK Parameters (Placebo Subjects)

	Placebo	
**Parameter**<br>Mean (SD)	**Imipenem**<br>n = 2	**Cilastatin**<br>n = 2
**F_e (%)**	36.766 (11.368)	120.359 (9.364)
**A_e (mg)**	183.832 (56.842)	601.795 (46.820)
**CL_r (mL/hour)**	4225.40 (1655.62)	12385.50 (420.44)

b. A_e: cumulative excretion of unchanged drug in urine; CL_r: urinary clearance; F_e: fraction excreted unchanged in urine; n: number of subject in the category; PK: pharmacokinetic; SD: standard deviation.

Table S4. Cohort 15: Urine PK Parameters (PK Parameters Population)

	Durlobactam		Sulbactam		Imipenem		
**Parameter**	**Day 1**	**Day 11**	**Day 1**	**Day 11**	**Day 1**	**Day 11**	**Day**
n (SD)	n = 10	n = 10	n = 10	n = 10	n = 10	n = 10	n = 1
**F_e (%)**	43.518 (11.601)	49.158 (18.278)	59.176 (13.995)	61.890 (18.383)	35.1762 (8.400)	54.343 (9.954)	88.37 (18.55
**A_e (mg)**	3481.42 (928.042)	491.58 (182.782)	4734.10 (1119.623)	618.896 (183.825)	1407.051 (335.998)	271.71 (49.770)	3535. (742.0
**CL_r (mL/hour)**	NC	NC	NC	NC	NC	8184.04[a] (2381.940)	NC

A_e: cumulative excretion of unchanged drug in urine; CL_r: urinary clearance; F_e: fraction excreted unchanged in urine; n: number of subject in the category; NC: not countable; PK: pharmacokinetic; SD: standard deviation.

a. n = 9.

Table S5. Study Design

Part A – Single Ascending Dose (n=6 DUR, n=2 placebo)
Cohort 1: single dose of 0.25 g IV DUR/placebo infused over 3 hours
Cohort 2: single dose of 0.5 g IV DUR/placebo infused over 3 hours
Cohort 3: single dose of 1.0 g IV DUR/placebo infused over 3 hours
Cohort 4: single dose of 1.0 g IV DUR/placebo infused over 2 hours
Cohort 5: single dose of 2.0 g IV DUR/placebo infused over 3 hours
Cohort 6: single dose of 4.0 g IV DUR/placebo infused over 3 hours
Cohort 7: single dose of 8.0 g IV DUR/placebo infused over 3 hours
Cohort 8 (n=8 elderly subjects [≥ 65 years]): single dose of 1.0 g IV DUR/placebo infused over 3 hours

Part B – Multiple Ascending Dose (n=6 DUR, n=2 placebo)
Cohort 9: 0.25 g IV DUR/placebo infused over 3 hours q6h for 7 days and 1 dose on Day 8
Cohort 10: 0.5 g IV DUR/placebo infused over 3 hours q6h for 7 days and 1 dose on Day 8
Cohort 11: 1.0 g IV DUR/placebo infused over 3 hours q6h for 7 days and 1 dose on Day 8
Cohort 12: 2.0 g IV DUR/placebo infused over 3 hours q6h for 7 days and 1 dose on Day 8

Part C – Single Dose Drug Interaction
Cohort 13:
- Day 1 - 1.0 g IV DUR/placebo infused over 3 hours
- Day 3 - 1.0 g IV sulbactam infused over 3 hours
- Day 5 - 1.0 g IV DUR/placebo plus 1.0 g sulbactam infused over 3 hours at the same time

Cohort 14
- Day 1 - 1.0 g IV DUR/placebo infused over 3 hours
- Day 3 - 0.5 g IV imipenem/cilastatin infused over 30 minutes
- Day 5 - 1.0 g IV DUR/placebo over 3 hours plus 0.5 g imipenem/cilastatin over 30 minutes at the same time as DUR/placebo
- Day 8 - 1.0 g IV DUR/placebo plus 1.0 g sulbactam over 3 hours at the same time plus 0.5 g imipenem/cilastatin over 30 minutes at the same time as DUR/placebo

Part D – Multiple Dose Drug Interaction
